# Quantitative Analysis of Dynamic Association in Live Biological Fluorescent Samples

**DOI:** 10.1371/journal.pone.0094245

**Published:** 2014-04-11

**Authors:** Pekka Ruusuvuori, Lassi Paavolainen, Kalle Rutanen, Anita Mäki, Heikki Huttunen, Varpu Marjomäki

**Affiliations:** 1 Department of Signal Processing, Tampere University of Technology, Tampere, Finland; 2 Department of Biological and Environmental Science/Nanoscience Center, University of Jyväskylä, Jyväskylä, Finland; 3 Department of Mathematical Information Technology, University of Jyväskylä, Jyväskylä, Finland; 4 Department of Mathematics, Tampere University of Technology, Tampere, Finland; Baylor College of Medicine, United States of America

## Abstract

Determining vesicle localization and association in live microscopy may be challenging due to non-simultaneous imaging of rapidly moving objects with two excitation channels. Besides errors due to movement of objects, imaging may also introduce shifting between the image channels, and traditional colocalization methods cannot handle such situations. Our approach to quantifying the association between tagged proteins is to use an object-based method where the exact match of object locations is not assumed. Point-pattern matching provides a measure of correspondence between two point-sets under various changes between the sets. Thus, it can be used for robust quantitative analysis of vesicle association between image channels. Results for a large set of synthetic images shows that the novel association method based on point-pattern matching demonstrates robust capability to detect association of closely located vesicles in live cell-microscopy where traditional colocalization methods fail to produce results. In addition, the method outperforms compared Iterated Closest Points registration method. Results for fixed and live experimental data shows the association method to perform comparably to traditional methods in colocalization studies for fixed cells and to perform favorably in association studies for live cells.

## Introduction

Live cell-imaging in subcellular scale has revolutionized the way cells are studied in molecular cell biology. As microscopy and imaging devices have enabled efficient and accurate live cell-imaging in high resolution, the demand for automated image analysis and interpretation has become obvious. For example, tagging proteins with specific fluorescent stains enables studying various cell functions through detection of protein-specific cell organelles, provided that the fluorescence-signal captured in digital images can be accurately analyzed. The spatial pattern and location [1], [2] of the detected signal may reveal the cell function or role of proteins, and colocalization of tagged proteins is in particular of interest [3]. In cell biology, close association of cellular structures, such as vesicles, occurs, e.g., in situations when vesicle pathways follow similar tracks or when close association is meaningful and leads to possible fusion events. There are very few tools available to study association. Instead, there are several tools to study colocalization, represented by different-colored voxels occupying the same spatial location. Association may be defined by a chosen distance between the objects. If differently colored objects are frequently associated they may be considered to keep near each other over time and follow each other in the cell cytoplasm. Analysis of closely associated objects in fixed cells allows accurate analysis, without errors caused by the movement of the objects between subsequent imaging of different channels – provided that the channels are aligned. However, in a live-imaging setup the quantification of sudden and transient events is challenging [4], and live imaging is prone to such errors that depend on the speed of the imaging setup.

Live imaging of cytoplasmic vesicles that are elicited from the plasma membrane after e.g. growth-factor stimulation or integrin activation reveals important aspects of the trafficking and fate of these crucial cellular regulators. As the growth-factor receptors and integrins use similar signaling pathways and show mutual regulation of important cellular processes [5] it has become important to follow their movement in live cells. The dynamic nature and interaction of these pathways provides complexity and makes reliable interpretation of the imaging results a very challenging task.

**Figure 1:**
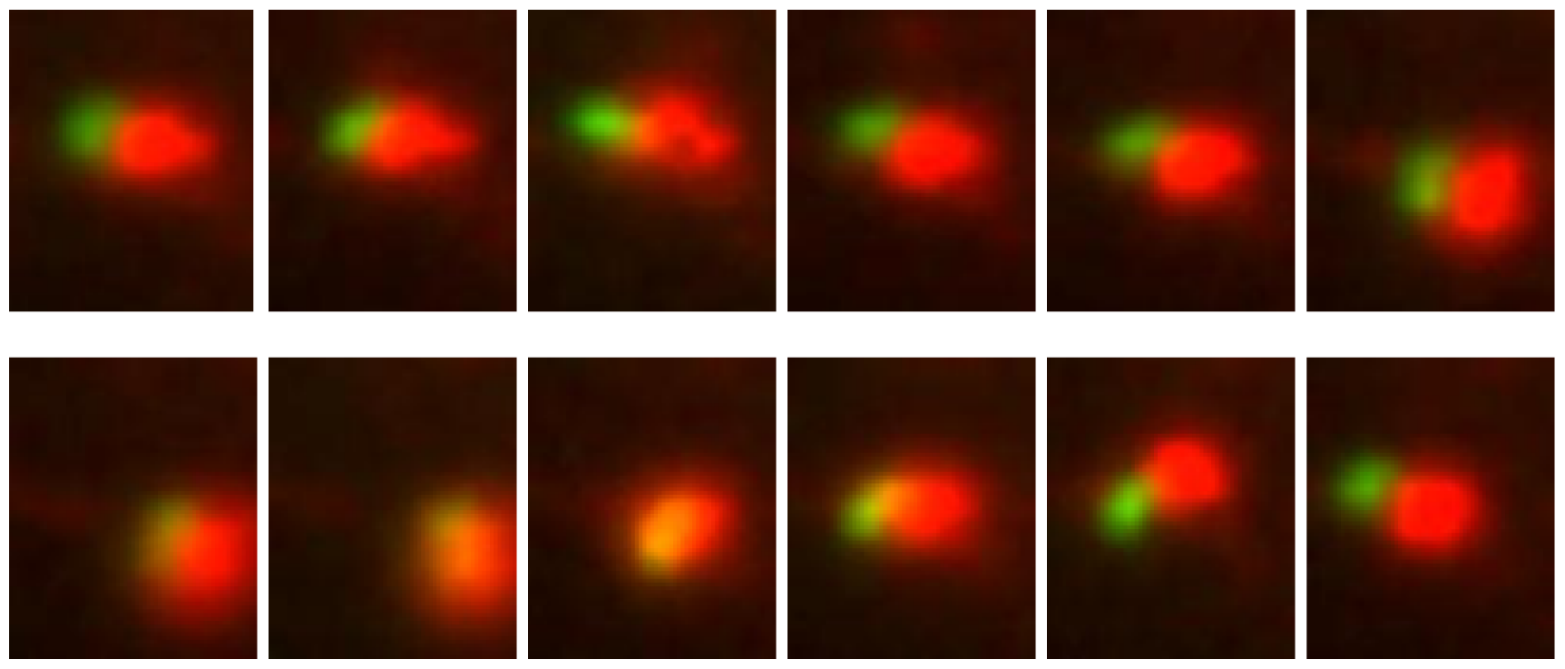
Close-up of a vesicle pair (red: integrin, green: EGF) in widefield microscopy images over a time-lapse. Time between successive frames is 5.7 s. Movement of vesicles in living cells causes situations where determining true colocalization may be ambiguous – colocalization determined as a direct overlap potentially misses close association of rapidly moving vesicles, and on the contrary, association determined using any method using objects in the proximity may give false detections due to closely located vesicles.

**Figure 2:**
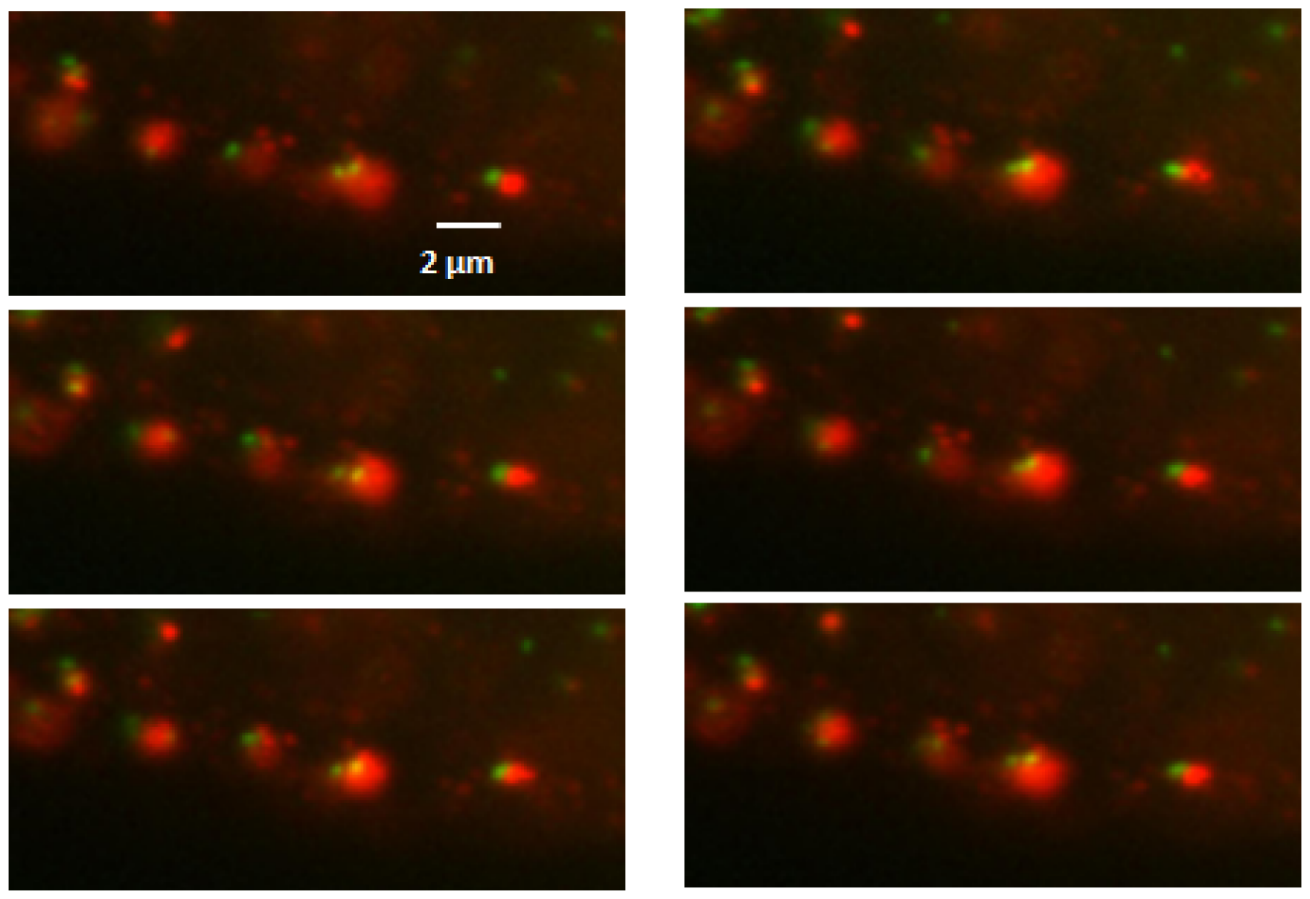
Close-up of a set of vesicles in live cell widefield microscopy experiment imaged over time (time between successive frames is 5.7 s). Low contrast and blur makes object detection challenging.

Traditionally colocalization analysis has been a subjective process, performed as a visual comparison of overlapping signal in two channels. Recent increase of the amount of image data and need for statistical analysis have shifted colocalization towards a more quantitative analysis. In many cases, colocalization is only partial leaving some voxels close by suggesting that fusion between the two colors has been meaningful but not complete. The partial colocalization may also indicate compartmentalization inside the structures. Therefore, instead of just colocalization, one could measure association with both voxels in close proximity and, in live images, voxels moving as one unit even if not entirely colocalizing. One example case where determining true colocalization may be ambiguous is given in Fig. 1 where a single structure or a pair of vesicle-like structures in close association is shown, and in Fig. 2 where the same vesicle is shown in a larger context with several similar structure pairs in two fluorescence channels.

One of the most straightforward quantitative methods include the pixel or voxelwise analysis where intensity of each element in an image channel is plotted against its counterpart in the other channel. This method, accompanied with statistical analysis of the significance of colocalization [3], [6] remains as one of the most common ways to estimate colocalization. Typically the studied biological phenomenon is related to specific subcellular organelles that have been labeled. Thus, instead of observing all the pixels or voxels containing signal it may be of interest to concentrate on the detected spots. Since the imaging resolution enables detection of individual organelles, it is possible to determine a quantitative estimate of colocalization objectwise [7]–[10]. Such approach makes it possible to take into account small changes in image due to imaging lag or other errors in the imaging phase. This can be advantageous in live cell-microscopy, where particle trafficking may be fast, compromising the accuracy of pixelwise colocalization analysis.

In this article, we propose a new approach to quantification of colocalizing or associating objects. The essential idea of the proposed method is to determine the mapping between images such that objects found in an image are paired with objects found in the compared image. Such problem is commonly addressed in dynamic monitoring of subcellular objects using live imaging where fluorescence-tagged organelles are followed throughout the imaging sequence [11]–[13]. However, to the best of our knowledge, matching of point sets using two channels of only one timepoint has not been proposed before for studying protein localization in fluorescence microscopy. Although both analyses rely on the property that the same target is imaged, colocalization or association analysis differs from live cell particle tracking in the assumption that the same objects may not be visible in the compared image. Also limiting to still images at sparse time resolution hinders the use of tracking methods to determine the mapping between frames.

Matching unpaired point-sets under a given class of transformations is a problem which can be divided into two main classes. In what is often called registration, a transformation close to an optimum matching transformation is known beforehand, and the problem is to refine that transformation to a nearby local optimum. By the prior information this local optimum is then also hoped to be close to a global optimum. In contrast, in point-pattern matching (PPM) nothing is known about the position of the global optima. Registration is local non-linear optimization, while PPM is global non-linear optimization.

In general, any point-set matching algorithm could be used as a basis of the proposed association analysis. Here we design an algorithm, based on PPM, particularly suited for the analysis of fluorescence microscopy images. PPM algorithms can be designed to be robust against changes in image geometry, e.g., changes in the number of detected particles, scale or shifts in image. In our application, the number of detected particles can typically vary significantly, whereas large systematic changes such as rotation or scaling between corresponding point locations are not expected. Thus, we may limit to translations and allow the method to only accept the transformation when the point sets are close to each other. Such small changes can be compensated by using PPM, however, they may cause the objects to be a miss in traditional co-localization analysis where co-localization is determined by pixelwise comparison of channels.

As an alternative way of finding point-pair correspondense, we use the Iterated Closest Points (ICP) [14] [15], or perhaps better described by Iterated Corresponding Points, which is a popular class of algorithms for solving the registration problem. There are many algorithms in this class, many of which are reviewed in [16]. An important trend in ICP-based algorithms has been to make them robust to missing/extraneous points (subset matching) and noise. As practical variants of this type, we mention the Biunique ICP [17] and the Trimmed ICP [18]. We provide a comparison to the Biunique ICP algorithm by making its initial transformation match the centroids of the point-sets; such an assumption may or may not hold for given microscopic images.

In this study we propose a novel method for association analysis. We show that the method is robust against moderate translations and object movement between image channels. The applicability of the method was demonstrated by following the entry of 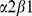 integrin and epidermal growth-factor receptor (EGFR) after triggering their internalization from the plasma membrane using fluorescent antibodies and fluorescent growth-factor, respectively. The results indicate that the vesicles containing 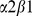 integrin and EGF are very close to each other along their internalization pathway to the center of the cell. Quantitative comparison between the proposed PPM-based association algorithm and the traditional colocalization estimates suggests that the results are in line for fixed cell experiments, with the new method providing improved detection of association by closely located objects in live cell experiments.

## Materials and Methods

### Microscopy data and live cell-imaging

The A549 (human lung carcinoma) cell line (ATCC) was used in all experiments. The cells were grown in Dulbeccos modified Eagle Medium (DMEM; Gibco) supplemented with 10% inactivated fetal calf serum (FCS), L-glutamine and penicillin-streptomycin (Gibco BRL, Paisley, UK) at 37°C, in 5% CO2. Cells were plated on cell culture chambers (Ibidi 15 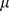-slide 8-well) two days before and starved (serum-free DMEM) for the last 16 hours before the experiment.

EGFR was stimulated and followed by microscopy by adding first biotinylated EGF (0,5 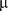
g/ml) on ice for 45 min and washed extensively. Then streptavidin Alexa 488 (5 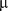
g/ml) was incubated on ice for 45 min and washed. 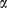
2
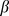
1 integrin clustering was done as described previously [19]. Briefly, specific antibody (A211E10; a kind gift from Dr. Fedor Berditchevski, Institute of Cancer studies, Birmingham, United Kingdom) against integrin was bound for 45 min on ice. Cells were washed extensively and incubated with a clustering secondary antibody (goat anti-mouse Alexa 594; Invitrogen). After washing, the cells were incubated in serum-free DMEM at 37°C to allow internalization. In practice, biotinylated EGF and integrin antibody was added simultaneously on cells and washed, and subsequently the fluorescent conjugates were added together on ice. The Ibidi slides were transferred to Zeiss Cell Observer HS (37°C, 5% CO2). The live imaging was performed using Colibri LED light source at 470 (41%) and 590 (46%) wavelengths for Alexa 488 and 594, respectively. Videos with 5.77 seconds (Live I), and 5.16 seconds (Live II) intervals were taken. In Figs. 1 and 2, close-ups of vesicles imaged using the maximum speed are illustrated.

For control experiments imaging perfectly colocalizing intensities, 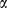
2
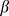
1 integrin clustering was induced using similar amounts of two fluorescent conjugates (goat anti-mouse Alexa 488 and 594). In addition, control videos of stable, non-moving vesicles were imaged after cells were fixed with 4% PFA for 20 min. For comparison, quantification of colocalization was determined using colocalization algorithms embedded in the free, open source software package, BioImageXD (http://www.bioimagexd.net, [20]). Only high intensity integrin clusters were selected for the colocalization analysis by first removing the background by subtracting the most common intensity value from the images. Next, masks for colocalization analysis were defined by filtering images with Difference of Gaussian (DoG) filter and subsequent thresholding. Small particles (less than 3 pixels for fixed and 8 pixels for noisier live cells) were removed from the masks in order to limit the detection of noise or small debris as spots. Finally, the masks were used for excluding background from the colocalization analysis.

### Simulated data

One of the motivations for using a point-set-based method for determining association stems from the fact that the image channels may be shifted or aligned non-ideally during the measurement process. The measurement consists of two separate image acquisitions with different filters applied in order to capture the desired wavelengths, corresponding to the specific fluorescent protein markers. To illustrate the robustness against such misalignments, we generated image sets with varying levels of global displacement in 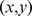 space, and an additional random movement term for individual spots. The experiment can be considered as a simulated live cell experiment, where the effect of potential displacement and movement due to imaging delay can be studied in a controlled manner. Importantly, the simulated experiments allow us to study the matching accuracy directly through examining the correct matches, mismatches and missing pairings. Thus, the simulations can be used both for evaluating the usefulness of the point-set based association analysis and for quantitatively comparing our PPM algorithm with a state-of-the-art registration algorithm.

Simulated experiments were generated as images with additive background noise and spots with varying intensity as foreground objects similarly as in [13]. The simulation parameters, *i.e.*, the number of objects, object size, and intensity, were inferred from real data in order to generate data that resembles realistic experimental image data. The key parameters of the simulation process were varied as follows. A global translation in random direction between the image channels was added using parameter space 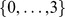 (in pixels), where 0 corresponds to no global shift, and 3 corresponds to maximum allowed magnitude of global translation. The other key parameter controls the random movement of individual objects. The movement was implemented as an additive random term drawn from zero-mean normal distribution 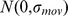, where 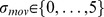 defines the magnitude of movement as deviation (in pixels) around the coordinate point. In the simulated images, the pixel size was set to correspond to 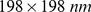. Furthermore, we generated three scenarios corresponding to low, intermediate, and high levels of association between channels 1 and 2, where the association levels with respect to channel 1 were set as 0.7477 in high, 0.5225 in intermediate, and 0.2072 in low association scenario. For channel 2, the association levels were set to 0.8557 in high, 0.4715 in intermediate, and 0.2805 in low association scenario. Number of objects was set to vary around a fixed number of 111 objects per channel, and the association levels between channels were controlled by adding and removing objects from random locations such that exactly the pre-set association levels were obtained. Finally, the simulations were replicated 10 times for each parameter settings. To summarize, the simulation study consisted of 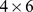 parameter combinations repeated in three association scenarios, each replicated 10-fold, resulting to 720 images with two channels. Object locations were extracted from the images using the DoG spot detection as described earlier.

### Determining vesicle association using colocalization analysis

Analysis of vesicle localization between two fluorescence-labeled image channels can be done in 2D or 3D. The true geometry of the samples means the 3D imaging with confocal microscope and subsequent processing in 3D enables more accurate analysis in theory. However, in live imaging such setting is not always applicable, since 3D imaging is time consuming whereas intracellular trafficking and object movement may be fast. In addition, use of 2D imaging enables higher throughput making 2D images a commonly used compromise in live colocalization studies. Thus, we will use 2D images taken in time-lapse live cell-imaging settings.

Some of the most widely used automated statistical colocalization estimates rely on correlation of the pixel intensities between the image channels. Here we use two pixelwise colocalization estimates, Pearson correlation and Manders' coefficient [21], as reference methods for comparison purposes. Pearson's correlation 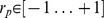 between channels 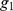 and 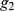 is given as
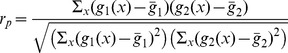
(1)where 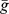 denotes the channel mean intensity. Positive correlation indicates match between channel intensities and suggests there exists colocalization of some level, whereas values close to zero show no correlation and thus give no evidence of colocalization. Possible negative correlations would indicate a negative relation of pixel intensities between compared channels. Another well-known statistical measure of pixelwise colocalization is the Manders' colocalization coefficient 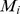, which is defined for channel 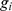 as
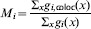
(2)where the colocalized proportion of the signal 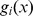 is given by
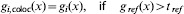
(3)where the selection of the threshold value 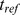 for the reference channel is essential in determining the colocalizing signal. Threshold selection, however, is not trivial and despite automated methods are available [3], [22] the selection may sometimes need to be adjusted by user. Recently, a colocalization measure combining both 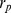 and 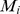 has been proposed in [23]. Here we have used the masks presented earlier to define the region of interest for the quantitative colocalization estimators. The results by both pixelwise estimators are obtained using the implementation available in BioImageXD.

### Vesicle association analysis using point-pattern matching

Our approach to determining association is to quantify the number of matching counterparts from two point sets after using point-pattern matching for aligning the point sets and for defining the matches. This approach could be implemented by using any suitable point set matching algorithm. However, here we define a point-pattern matching algorithm which makes use of the properties of the application area. The PPM algorithm described here, and an alternative point set matching algorithm using ICP, can both be tested by using the implementations available on our supplementary site.

Given two finite sets of points, say 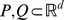, and a set 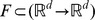 of allowed transformations, a PPM algorithm attempts to determine a transformation 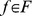 such that at least some subset of the points in 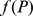 would match some subset of 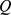. In this paper we will fix 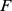 as the set of translations, i.e. each 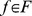 is of the form
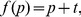
(4)for some 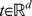.

The term match must be defined carefully to obtain meaningful results from a PPM algorithm. For example, defining a match between 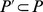 and 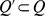 by the relation 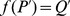 means that a possible match is destroyed by an arbitrary small translation to any point in either 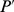 or 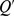. Since any real-world measurement contains noise, a practical PPM algorithm needs to be able to maintain a match under small deviations of the point-sets, i.e. to be robust under noise. In addition, since in practice some measurements can be missing or extraneous in either 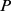 or 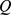, a practical PPM algorithm should be able to find matches between subsets 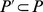 and 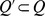 also.

Considering the application area, if we are to apply PPM to determine colocalization, then the need for both kinds of robustness is seen as follows. First, it is likely that the detected object-sets do not match perfectly even in the case of nearly perfect colocalization, since the measurements are from different objects. Second, differences originate from biological variation which leads to varying levels of colocalizing points. Third, even when the targeted objects are colocalized, the point-sets include “noise” from object movements.

The way the quality of a matching is measured affects the robustness of a PPM algorithm tremendously. For example, if there were an exact copy of the model point-set (what to find) in the scene point-set (from where to find), but there were an additional distant cluster of points, we would like the cluster to not affect the matching result. Many papers on PPM concentrate on minimizing the Hausdorff distance between point-sets [24] [25] [26], defined as
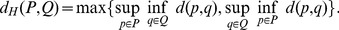
(5)Unfortunately, this distance can be made arbitrarily large by introducing an additional distant point in either 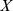 or 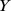. For this reason, we reject the minimization of Hausdorff distance as a practical matching strategy. To improve on the robustness issue, several authors have proposed using partial Hausdorff distance instead, where the supremum is taken only over a given percentage of the smallest distance values. By doing this, it is hoped that the procedure correctly rejects any points that are too far away to be meaningful for the matching. Unfortunately, no percentage is small enough to guarantee that such outliers are correctly rejected; the number of additional distant points can always be increased so that the ratio of outliers exceeds the given percentage. For this reason, we also reject the minimization of the partial Hausdorff distance as a practical matching strategy.

Instead, we adopt the matching criterion from [27]. Intuitively, a point 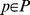 matches a point 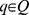 under 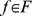, if 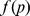 is close to 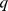. By extension, 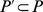 matches 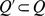, if each point in 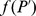 has a unique match in 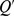. This intuition is made exact in the next section where we formulate the matching algorithm. Our approach is to find a match under a given pointwise matching distance, and then determine the amount of association from this correspondence.

Van Wamelen et al. [27] presented a fast algorithm for PPM in 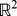 under conformal affine transformations, with a robust matching criterion which we adopt. We do not, however, select this algorithm, because the application we are dealing with requires a high degree of robustness. Moreover, the algorithm can fail on given parameters, for which there is no systematic way to set to suitable values beforehand. In fact, the requirement for finding a match if such exists sets a limit for possible algorithms, and we are not aware of existing studies that would fit our application. Instead, we will construct one in the next section.

### Point-pattern matching under translations

In the following we present an algorithm for PPM between two finite point-sets in 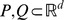, 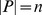, 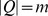, when the class of transformations 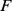 is given by translations 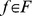 which align 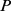 to 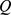:
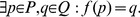
(6)The algorithm either reports that there is no match, or reports a bijection between subsets 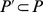 and 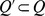, such that 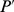 and 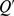 match.

### Matching criterion

Let 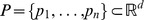 and 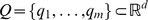 be two sets of points. Let 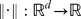 be a norm in 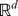. Given 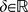, 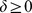, called the matching distance, and 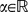, 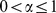, called the matching ratio, the point-set 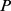 is said to *match* the point-set 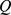 if there is a set 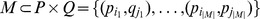, called a matching, where each point in 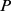 and 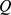 is part of at most one pair,

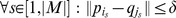
, and

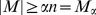
.

In addition to the matching criteria above, we also set a limit for the bias of a match, which will be discussed next.

### Bias of a matching

Even if we find a matching 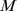, it might be that the matching is of poor quality. Assume that for a translation 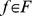 it holds that 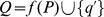, where 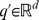, and it holds that 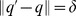 for some 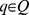. That is, 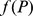 matches 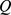 perfectly, but there is an extraneous point 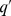 nearby 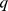. Let 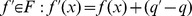. Then also 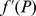 matches 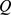. However, the matching given by 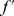 is of poor quality because the difference vectors between the pairs in the matching are all (except one) in the same direction. We would rather want the error to be distributed uniformly in all directions. To avoid these systematically poor matchings, we define the bias of a matching *M* by
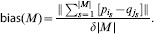
(7)We will then define a maximum allowed bias 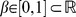, and require from a matching that 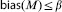. The matching provided by 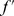 in the example can then be avoided, by choosing 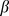 properly, since the errors average near to zero with 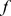 and near to one with 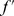.

### Nearest neighbors searching

In nearest neighbors searching, the problem is to report those 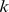 points in 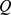 which are closest to a given point 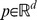. To search for the 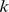 nearest neighbors in 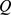, we shall use the kd-tree data structure with the sliding midpoint splitting rule [28]. This data structure can be constructed for 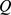 in 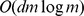 and 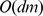 space.

In the following we shall assume that for each nearest neighbors search there will not be two points in 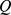 with the same distance to 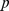. This simplification is without loss of generality; in an actual implementation one gives a secondary order to equidistant points, for example, by giving them an ordered labeling. Let 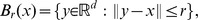 where 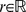 and 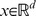.

### Maximum bipartite matching

Let 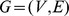 be a graph (all graphs in this paper are directed and simple), where 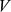 is a finite set of vertices, and 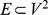 is a finite set of edges. A graph is called bipartite, if it can be decomposed as 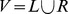 with 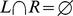, and 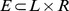. A matching of *G* is a subset 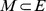 without common vertices (this definition is consistent with the matching of point-sets after we define 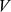 and 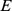 in the algorithm). A maximum matching of *G* is a matching of 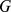 with the largest possible number of edges 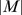. We use the Hopkroft-Karp algorithm [29] to compute a maximum matching in a bipartite graph in 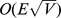 worst case time, or in 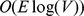 average time for random graphs.

### PPM Algorithm

Assume a matching distance 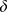, a matching ratio 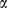, a maximum allowed bias 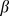, and a maximum number of nearest neighbors 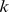. The PPM algorithm consists of repeating the following steps for each pair 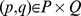.

1. Let 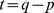.
2. For each 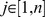, find 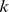 (or as many as possible) nearest points 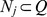 to 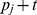 in 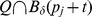.
3. Let 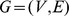 be a (bipartite) graph, where 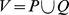, and 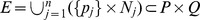.
4. Find a maximum bipartite matching 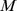 in 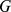.
5. If 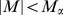, start with a new pair 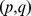.
6. If 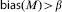, start with a new pair 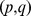.
7. Return 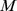 as a matching between 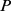 and 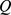.

### Object-based association analysis using point-pattern matching

In this section we apply the above PPM algorithm for estimating protein association between image channels. First, since we are using PPM, the fundamental requirement is to obtain point-sets where the points denote fluorescence-labeled objects detected from the image. These objects, appearing as resolution-limited, low contrast blurry spots are challenging to extract, but methods for detection have been presented and evaluated in the literature [30], [31]. Here we leave the discussion about the selection of detection algorithm out of the scope and note that we made the method selection based on experimenting and used the method described earlier for detecting the fluorescent objects, and the centroids of detected objects from two channels form the point-sets 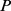 and 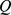.

**Figure 3:**
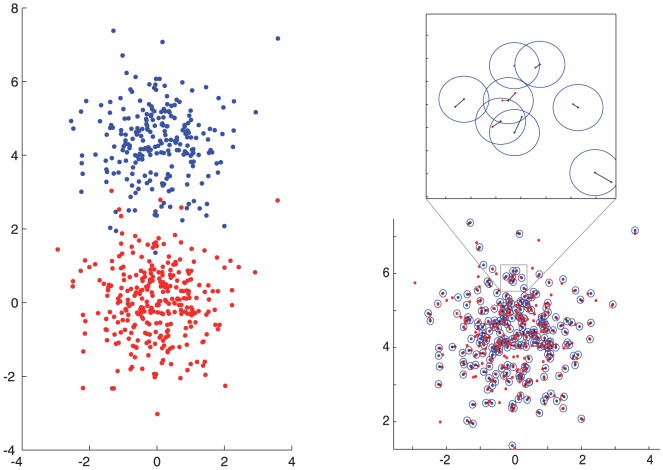
Example of matching point-sets. Left: Point-set (red) and an altered set (blue) under noise, transformation, and with missing points with probability of 0.2. Right: The same point-sets after matching. Each matched point has been marked with a line to the corresponding point in the other set. The search area has been shown with circles. Top: Close up where matches and search areas can be seen.

Given the PPM algorithm under translations described above, it is now possible to define the similarity between point-sets by finding a transformation between 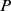 and 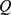. This transformation gives us the following information. First, if a match cannot be found, there is no association at the specified level of correspondence, which is given as the matching ratio 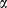. Second, if a match has been found, we can check the transformation in order to find out how much the point coordinates had to be altered in order to find a match. A moderate transform suggests that true association exists at the matching ratio 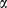, whereas a drastic transformation tells about a correspondence found by chance which should not be counted as association. Using these observations, a rule for estimating association under the restrictions can be defined by
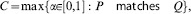
(8)where 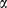 is the matching ratio, and the matching criterion is as defined previously. While 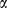 is a real number, the matching algorithms only differ on finitely many values of 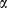, corresponding to the different number of required points 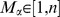 in a matching. The values of 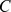 in Eq. 8 can be interpreted similarly as, for example, direct pixel or objectwise overlap values – values close to the maximum value of 1 correspond to high level of association and values close to the minimum value 0 mean there is very little association – with the only difference being that also closely located objects are allowed and direct overlap is not required in the case of the PPM-based association. Fig. 3 illustrates an example matching using simulated data with 300 points drawn from normal distribution and 0.2 ratio of missing points between channels, and with translation and noise introduced for the point-set. The matches are marked with lines in the close-up, and circles illustrate the search range defined with parameter 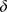.

### Notes on implementation and 3D

Using two-dimensional projections as a basis of association analysis is a choice made for practical reasons. However, cells are naturally 3D objects, and thus confocal microscopy suits well for true 3D colocalization studies. In such cases, the objects can also be extracted in 3D, yielding an additional location coordinate. Although 2D imaging is used in the experiments of this article, estimating association with the new method based on PPM is also possible in 3D. Majority of the computational workload comes from the matching process, thus, we implemented the PPM algorithm in C++. The implementation is available from supplemental site https://sites.google.com/site/vesicleassociation/.

## Results

We present quantitative results for both simulated and real data. Simulated data, for which ground truth is available, is designed for demonstrating the properties and validating the performance of the PPM-based association algorithm. Importantly, simulation serves as a powerful tool for validating the novel approach based on point-set matching, and it enables comparing the proposed matching algorithm to state-of-the-art algorithm directly, using matching accuracy as the criterion. Real data, collected using experimental setup described in Materials and Methods section, allows comparison with traditional colocalization measures in true use cases. Due to the lack of ground truth only indirect measures of accuracy can be used, as is typically the case for real data. The real experiments, however, can be validated through the biological setup. We have used two different scenarios; fixed cells with very high level of colocalization between the labeled structures and live cell experiments with known association of labeled structures without a perfect overlap.

The results section starts by an extensive simulation study where we demonstrate the properties of the PPM-based association algorithm and compare it with ICP matching. Furthermore, we show how the proposed method is able to detect associations under circumstances where traditional colocalization estimates fail to produce accurate results. Second, we present a comparison with fixed cells where traditional colocalization measures are known to perform well. Third, we show how the association algorithm performs in live cell experiments and again compare against traditional colocalization methods. Finally, we demonstrate robustness to artificially generated imaging delay between frames in live cell experiments.

### Robustness to channel displacement and object movement with simulated data

One of the key advantages in simulation is that the ground truth, that is, the correspondence between objects in the two channels is known. This enables the use of quantitative measures of matching accuracy instead of evaluating the results only based on the association estimate. Here we use the ground truth information for determining true positive matches (object paired with a correct counterpart), false positive matches (mismatch, object paired with a wrong counterpart) and false negative matches (object not paired although counterpart exists), from which the precision, recall, and subsequently, the F-measure are determined as explained in [32].

**Figure 4:**
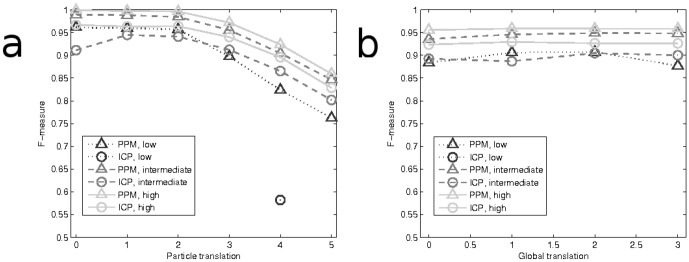
Result summary for simulated experiments. Results for PPM (triangle) and ICP (circle) are given as average F-measure for ten replicate simulations for each parameter combination. All three simulation scenarios are shown in the same figure; high association level with solid line and light grey, intermediate association level with mid grey dash line, and low association level with dotted dark grey line. (a) Results illustrated with standard deviation of the random particle movement (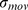) as a parameter. (b) Results illustrated with length of the global transformation (in pixels) as a parameter. F-measure is calculated through quantifying true and false matches, and results are not shown for parameter combinations leading to undefined F-measures.

In Fig. 4, the F-measure results by PPM and ICP are shown as summaries across all 10 replicates, and parameter combinations leading to undefined F-measure (due to failed matching where none on the pairings were true positives) were left out of the graph. The results for both algorithms are obtained with matching distance 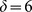, which corresponds to roughly 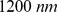 distance with the simulated data. Further, we used the simulation experiment for studying the effect of the matching distance parameter in both point-set based algorithms (Supplemental results) without significant changes in the relative performances.

The results in Fig. 4 confirm that the point-set based approach for determining object association is able to handle moderate object movements (Fig. 4 (a)) and that global translation between image channels can be eliminated very efficiently (Fig. 4 (b)). When examining the results given by the two matching algorithms, it can be seen that PPM slightly but consistently outperforms the ICP method for high and intermediate association values. Moreover, the results confirm that, as can be expected, both of the methods relying on point-set matching generally perform more accurately when the level of association is higher, but the drop in matching accuracy due to lowering association level from approximately 0.80 to 0.25 was not dramatic for the PPM, whereas the ICP-based method resulted to several failed pairings with low association values (leading to missing values in the graphs). Given that it is common in this application that the association level may be low either due to the lack of association in the studied biological phenomenon, or due to severe imbalance in the number of objects in the channels, we will conclude based on the presented results that the proposed PPM-based method should be preferred for matching the point sets. In the remaining experiments, we will only use PPM for representing the proposed point-set based approach.

### Comparison with traditional colocalization estimates with fixed cells

Second, we estimate colocalization for fixed samples from human lung carcinoma cells. This data can be considered as a reference set since the cells are fixed and 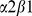 integrin was labeled using similar amounts of two fluorescent conjugates inducing almost perfect colocalization. The quantitative results for four sets of fixed cells (denoted by Fix I–IV) comprising 277 images with two fluorescence channels are shown in Table 1. For PPM, we used matching distance 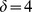, and maximum bias 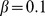. The matching distance was determined through setting a limit for the allowed area of determining association using expert knowledge and information about the pixel dimensions; here 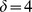 corresponds to 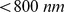. As expected, the association results given by the PPM-based method are rather high which is well in accordance with the experimental setup.

**Table 1:** Colocalization and association estimates for fixed cell image sets.

Image set	#images	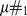	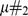	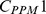	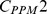	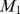	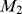	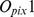	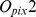	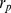
Fix I	71	154.41	126.46	0.6719	0.8123	0.7198	0.8111	0.6407	0.7300	0.7008
Fix II	70	154.40	67.37	0.4168	0.9493	0.4938	0.8946	0.3970	0.8469	0.7166
Fix III	69	201.32	82.06	0.3844	0.9345	0.4994	0.8705	0.3419	0.8266	0.7882
Fix IV	67	92.45	71.73	0.5645	0.7079	0.5441	0.7525	0.4784	0.6310	0.8116

Average number of objects per channel for each set are given as 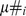, association estimate by the PPM-based methods in 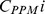, Manders' colocalization coefficient is 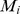, pixelwise overlap is 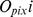, and 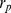 is the Pearson correlation.

For comparison, we estimated the colocalization by the commonly used Pearson correlation (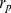) as well as with the Manders' colocalization coefficient (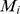) where the DoG method was used for masking as explained earlier, and 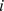 refers to the image channel. Also the direct overlap percentage of pixels (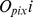) was calculated for both channels using the masked images. The results suggest that the PPM-based method yields colocalization estimates which in general behave similarly as the Manders' colocalization coefficient as well as the traditional pixelwise colocalization estimate. Importantly, allowing the point-set based method to determine association within the matching distance instead of limiting to direct colocalization does not seem to result to overestimated values when compared to the traditional colocalization measures.

### Association and colocalization in live cell-imaging

**Figure 5:**
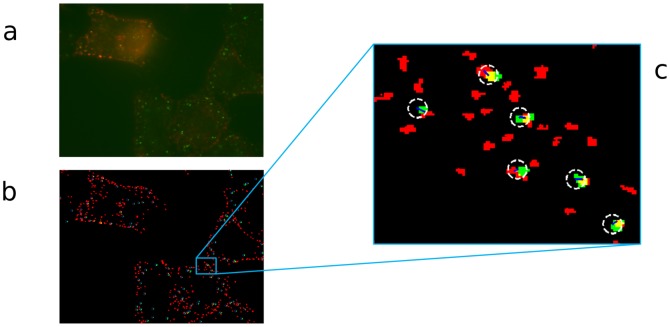
Frame from a live-cell imaging experiment with; a) overlay of original image channels, b) detected objects, colors correspond to image channels and direct pixelwise overlap (colocalization) is visible as yellow color, c) close-up showing transformed point set and search area with white dashed circles and found matches (association) with blue lines.

Next, we assess the PPM-based method in live microscopy where movement of vesicles potentially affects to traditional colocalization estimates. We use two live microscopy datasets with integrin labeled cells from the A549 cell line imaged at over 150 time-points. The labeled structures are now different, thus direct colocalization is expected to be low but the structures are known to be closely associating. An example image can be seen in Fig. 5 (a) and the objects detected from both channels are shown in (b). In total, there are 330 images with two fluorescence channels, and the average particle counts (size limited to be 8 pixels minimum) are given in Table 2. The PPM maximum bias was again set to 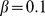, and matching distance was set to 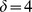 corresponding to 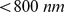 distance, which defines the allowed area for determining association. The matching process is visualized in Fig. 5 (c) where the matching area (defined by the matching distance) is shown with white circle around the transformed point locations, and paired objects are shown with blue lines connecting the object centers after applying the transformation by PPM algorithm.

**Table 2:** Colocalization and association estimates for live cell-imaging experiments.

Image set	#images	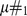	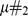	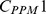	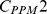	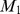	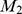	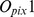	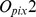	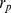
Live I	156	103.60	260.69	0.3813	0.1528	0.1618	0.0850	0.1595	0.0615	−0.0294
Live II	174	142.37	410.02	0.4682	0.1650	0.1574	0.0831	0.1690	0.0561	−0.0590

Average number of objects per channel for each set are given as 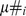, association estimate by the PPM-based methods in 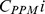, Manders' colocalization coefficient is 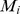, pixelwise overlap is 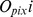, and 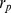 is the Pearson correlation.

In Table 2, the results for a live cell experiments are given. Based on the results, the traditional methods do not give much colocalization whereas the association estimate by PPM suggests that, even though the structures are not directly overlapping, there exists a certain level of association which is only revealed by the new point-set based method. The close-up area shown in Fig. 5 (c) shows examples of points located very closely in the two channels having very little or no direct overlap, but which are paired by the PPM algorithm, leading to detected association. This is in line with control colocalization measurements of 3D data in confocal microscopy [33] and shows a high amount of association but very limited direct colocalization. Results for the two datasets (denoted as Live I & II in Table 2) are almost identical. The data thus suggest that EGFR and integrin-positive structures stay close but separate after their triggered internalization. This has also been recently shown by us using confocal 3D colocalization analysis [33].

### Robustness to imaging delay with real data

**Figure 6:**
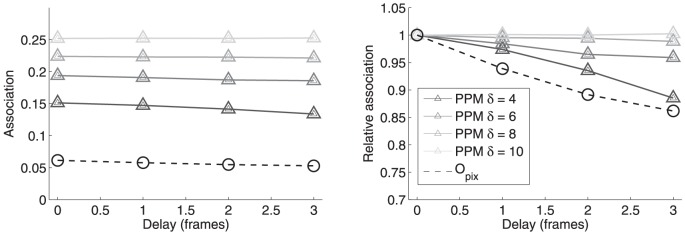
Effect of delay to the estimate. Delay is given in frames (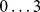). The left graph shows the numerical estimates for association by PPM using different parameter values for search range (grey graphs with triangle markers) and direct pixelwise colocalization (black graph with circle markers). On the right, the results are presented relative to the first datapoint, *i.e.* the values are divided by the estimates obtained for the first, non-delayed values.

The robustness of the algorithm against delay in imaging process is studied next. Different levels of delay are considered by using frames 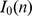 and 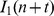 for determining colocalization, where 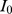 and 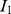 are the two image channels, 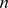 is the current frame and 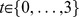 is the delay in frames, where the length of delay is defined by the imaging frequency (here the delay is multiples of 5.7 s). The results are shown in Fig. 6 both as numerical estimates and as relative values normalized by the first, non-delayed datapoint. The results are presented with respect to channel 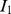. For PPM we evaluated the effect of search range parameter 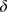 by giving values 4, 6, 8, 10. Given the rather long delay, this time it is justified to use larger values for 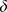. The objectwise overlap was calculated for comparison purposes using the same segmentation masks which were also used as a basis of PPM matching. The results represented as relative to the non-delayed case reveal how the direct pixelwise colocalization estimate and the PPM method with small search range are sensitive to imaging delay, whereas association values obtained with larger search ranges are less affected by the artificially introduced delay. The results also indicate that PPM-based association estimates are larger than direct overlap-based colocalization, as was expected due to the lack of direct colocalization of the labeled structures.

## Discussion

In this article, we have presented a computational method for estimating protein association between two image channels using an object-based point-pattern matching approach. The method searches a mapping between point sets detected from the image channels through pairing individual objects detected in both channels. The association is estimated as the fraction of paired objects, creating a measure that is directly comparable to the colocalization percentages between overlapping objects or masked image pixels/voxels. The advantage of the proposed method stems from the inherited robustness of PPM against directed movement between the image channels, which could be potentially caused by misaligned image channels. Also other moderate transformations, such as random object movement during the lag in the imaging of the fluorescence channels, can be compensated within the search area. Any movement of the fluorescence-labeled subcellular structures will potentially lead to missed colocalization unless the movement is compensated, whereas the proposed method based on PPM is able to resolve association in cases where moderate object movement exists. The robustness against moderate translation and object movement are beneficial in live cell imaging where rapid movement of objects occur, in such cases the use of PPM-based method provides an advantage over the traditional colocalization measures such as Pearson correlation or Manders' coefficient. Furthermore, the proposed method does not limit to quantification of direct colocalization, but it also detects the nearby objects associated with the same structure. The robustness of using the point-set matching comes at the cost of an additional computational step. Since determining colocalization or association is not typically a real-time task, this disadvantage does not hinder the use of the PPM-based method.

The proposed PPM-based method was experimentally validated with several time-lapse image sequences with hundreds of image frames, each image typically containing in the order of hundreds of fluorescence-labeled subcellular objects. The results obtained for simulations demonstrate the benefits of the proposed method. Misalignments and random movement of individual objects cause significant drop in the performance of traditional methods, whereas the matching based PPM and ICP methods performed well when the random movement was moderate. Moreover, the results obtained for fixed human lung carcinoma cells with known colocalization of two 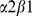 integrin fluorescent conjugates show that the estimates by the PPM association method are well in accordance with the traditionally applied pixelwise colocalization estimates, such as the Manders' colocalization coefficient and Pearson correlation, as well as with colocalization estimated with direct object-based overlap. Finally, we studied how association estimate performs in live cell experiments using live microscopy of 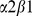 integrin and EGF labeled cells from the A549 cell line. It was shown that dynamic association of two structures which do not perfectly overlap could be detected by the proposed method. Further, the effect of delay on the colocalization in live imaging situations was demonstrated, and the results under artificially created heavy delay further confirm the robustness of our method compared to estimate without movement compensation. With these results, the PPM association method can be recommended for object association studies as well as a robust substitute for traditional colocalization methods for live biological fluorescent samples.
